# Plasma Septin9 versus Fecal Immunochemical Testing for Colorectal Cancer Screening: A Prospective Multicenter Study

**DOI:** 10.1371/journal.pone.0098238

**Published:** 2014-06-05

**Authors:** David A. Johnson, Robert L. Barclay, Klaus Mergener, Gunter Weiss, Thomas König, Jürgen Beck, Nicholas T. Potter

**Affiliations:** 1 Gastroenterology Division, Eastern VA Medical School, Norfolk, Virginia, United States of America; 2 Rockford Gastroenterology Associates, Ltd., Rockford, Illinois, United States of America; 3 Digestive Health Specialists, Tacoma, Washington, United States of America; 4 Epigenomics AG, Berlin Germany; 5 Molecular Pathology Laboratory Network, Inc., Maryville, Tennessee, United States of America; Sapporo Medical University, Japan

## Abstract

**Background:**

Screening improves outcomes related to colorectal cancer (CRC); however, suboptimal participation for available screening tests limits the full benefits of screening. Non-invasive screening using a blood based assay may potentially help reach the unscreened population.

**Objective:**

To compare the performance of a new Septin9 DNA methylation based blood test with a fecal immunochemical test (FIT) for CRC screening.

Design: In this trial, fecal and blood samples were obtained from enrolled patients. To compare test sensitivity for CRC, patients with screening identified colorectal cancer (n = 102) were enrolled and provided samples prior to surgery. To compare test specificity patients were enrolled prospectively (n = 199) and provided samples prior to bowel preparation for screening colonoscopy.

**Measurements:**

Plasma and fecal samples were analyzed using the Epi proColon and OC Fit-Check tests respectively.

**Results:**

For all samples, sensitivity for CRC detection was 73.3% (95% CI 63.9–80.9%) and 68.0% (95% CI 58.2–76.5%) for Septin9 and FIT, respectively. Specificity of the Epi proColon test was 81.5% (95% CI 75.5–86.3%) compared with 97.4% (95% CI 94.1–98.9%) for FIT. For paired samples, the sensitivity of the Epi proColon test (72.2% –95% CI 62.5–80.1%) was shown to be statistically non-inferior to FIT (68.0%–95% CI 58.2–76.5%). When test results for Epi proColon and FIT were combined, CRC detection was 88.7% at a specificity of 78.8%.

**Conclusions:**

At a sensitivity of 72%, the Epi proColon test is non- inferior to FIT for CRC detection, although at a lower specificity. With negative predictive values of 99.8%, both methods are identical in confirming the absence of CRC.

**Trial Registration:**

ClinicalTrials.gov NCT01580540

## Introduction

Colorectal cancer (CRC) remains a highly prevalent disease, with an estimated 143,000 new cases and 50,000 deaths anticipated in the United States in 2013 [1]. Early detection of CRC through screening of asymptomatic individuals decreases the mortality from CRC [2], [3], and CRC patients detected by screening generally have earlier stage cancer and a better prognosis [4]. Data from the American Cancer Society indicates that the 5 year survival rate for early stage CRC is >90%, and as high as 72% for Stage III cases [5]. Despite the good prognosis and the widespread availability of multiple options for CRC screening participation in screening programs remains suboptimal. Estimates suggest that over one third of adults in the US have not been screened for this disease [6], [7]. In fact, comparing the 2010 and 2012 statistics indicates that participation rates for CRC screening have leveled off at approximately 65%, indicating that reaching the goal of 80% set by the Centers For Disease Control may be difficult [7].

Providing a choice of tests was shown to positively impact participation in CRC screening [8]. However, currently available tests have limitations (e.g. fecal sampling, bowel preparation, procedural risks) that present significant hurdles to participation [9]. Blood based screening is routine and well accepted for many health conditions, and its availability for CRC screening could significantly increase participation rates. Methylated Septin9 DNA in plasma is the first biomarker developed and validated for this objective [10].

The *SEPT9* gene encodes Septin-9, a member of the conserved septin family of GTP-binding proteins that function in key processes including vesicle trafficking, apoptosis, cytoskeletal remodeling and cell division [11]. The Septin-9 protein also acts as a tumor suppressor, regulating orderly and controlled cell growth. Alterations in the activity/expression of the *SEPT9* gene have been associated with a number of cancers including breast, ovarian, prostate and colon [12]–[15]. The altered gene expression may also enhance cancer-related events including cell division, cell movement and angiogenesis [12]. The *SEPT9* gene has a complex promoter structure, and the specific sequence for which differential methylation is reported in CRC occurs in the gamma1 promoter region that is transcribed as part of the *SEPT9*_v2 transcript [16]–[18]. The methylation status of this sequence has been shown to discriminate CRC tissue from normal mucosa [17]–[19].

The clinical performance of the Septin9 marker has been validated in multiple studies with more than 5000 subjects. In these studies, sensitivity ranged from 69% to 95% and specificity ranged from 85% to 95% [10], [20]–[24]. In a prospective trial (PRESEPT NCT00696345) using a CE-marked kit for methylated Septin9 detection, 50.9% of cancers were detected at 91.5% specificity based on a two replicate PCR test. In an ad-hoc analysis using a triplicate PCR, 34 of 51 cancers (66.7%) were detected. Standardized against US population data, this yielded a clinical sensitivity of 64% at 88.4% specificity [24]. The Septin9 marker was developed into the Epi proColon test and evaluated in a case-control setting where the cancer sensitivity was 95% at a specificity of 85% [25]. In a prospective trial, cancer sensitivity was 68% at a specificity of 79% (unpublished data). The reference standard was colonoscopy and did not include comparisons to other approved modalities. Fecal occult blood tests (gFOBT/iFOBT) are currently the only non-invasive tests recommended under all US screening guidelines and are an important comparator for the performance of the test.

With this report, using colonoscopy as the reference standard, we compared the performance for CRC detection of Epi proColon and the immunochemical fecal occult blood test (OC FIT-CHEK Polymedco; Cortland Manor NY).

## Methods

The protocol for this trial and supporting TREND checklist are available as supporting information; see Checklist S1 and Protocol S1.

### Patient Samples

Institutional approval for 61 sites in the United States was granted by Western IRB (WIRB). Local approval was also granted at Middlesex Hospital IRB, Biomedical Research Alliance of New York (BRANY) IRB, Dean Foundation IRB, Beaumont Hospitals IRB/HIC, The Mary Imogene Bassett IRB and HCA Midwest Health System IRB. Written informed consent was obtained from all study subjects prior to enrollment. The first subject was enrolled on March 30^th^, 2012 and the final procedure was completed on November 26^th^, 2012. The trial was registered in clinicaltrials.gov on April 17^th^, 2012 (NCT01580540). Although this was after first enrollment due to an oversight on the part of the sponsor, it was within the required 21 days. The authors confirm that all ongoing and related trials for this drug/intervention are registered.

Eligible subjects were recruited from individuals scheduled to undergo screening colonoscopy or from patients who had been diagnosed with CRC through screening colonoscopy. All colonoscopies were performed by board certified gastroenterologists. Although the quality of the colonoscopies performed in this trial was not tracked in a detailed fashion, adequacy of bowel preparation and completion of the colonoscopy (visualization of the cecum) were required per study protocol. The observed rate of 39% for small polyps and 13% for advanced adenomas in the prospective arm of the trial supports the high quality of the colonoscopies performed in the study.

Individuals aged 50–84 years were enrolled in the study. Exclusion criteria included a previous history of CRC or previous colonoscopy resulting in recommendation for repeat colonoscopy at an interval less than ten years (high risk population); neoadjuvant treatment; familial history of CRC; history of inflammatory disease; acute or chronic gastritis; current diagnosis of cancer other than CRC; overt rectal bleeding or bleeding hemorrhoids; known infection with HIV, HBV or HCV; and receiving intravenous fluid at the time of the sample collection. Subjects with a curative biopsy during screening colonoscopy were also not included.

Information collected comprised age, gender, height, weight, and ethnicity; diagnostic information related to pathological confirmation and staging of CRC for the screening identified CRC cases; and the number, size, and location of polyps for the prospective screening subjects. Information on date and time of blood draw, centrifugation, and plasma handling was collected and the date of stool sample collection and FIT testing.

Based on the results of a complete colonoscopy (defined as having adequate bowel prep and reaching the cecum) non-CRC subjects were classified as follows : Small Polyps (SP) - subjects had a polyp(s) <10mm in size having no evidence of high grade dysplasia and no villous component; Advanced Adenoma (AA) – subjects had a large adenoma(s) (≥10 mm) and/or had lesions(s) with a villous component, and/or had lesion(s) with high grade dysplasia; No Evidence of Disease (NED) - subjects had no evidence of CRC, high grade dysplasia, advanced adenomas or small polyps.

### Study Design

This prospective multicenter study was designed to collect matched blood and stool specimens from screening guideline-eligible subjects. The primary objective was to compare the clinical performance in terms of test positivity of the investigational use only (IUO) Epi proColon test to the OC FIT-CHEK test. The study population was enriched by enrolling subjects with screen-detected CRC or high suspicion of CRC for the sensitivity comparison (Group A). Prospectively collected screening subjects provided a sufficient sample size to perform a specificity comparison (Group B).

### Specimen Processing

Blood samples were obtained from each subject, processed and aliquoted according to the instructions for use for the Epi proColon test. The test kits were produced under Good Manufacturing Practice (GMP) and met specified performance criteria. The plasma aliquots were shipped frozen to a central repository and archived at −80°C for later testing. Stool samples were collected at home by the subjects with provided kits following the manufacturers’ instructions; samples were shipped directly to the testing laboratory. Group A subjects were required to have had a colonoscopy within 6 months, and provided blood and stool samples a minimum of 10 days following colonoscopy, but prior to resection surgery. Group B subjects provided blood and stool samples prior to bowel preparation for their screening colonoscopy.

The FIT testing was performed for the OC-FIT CHEK samples using an OC-Auto analyzer (Polymedco; Cortland Manor NY) which has a 100 ng/mL hemoglobin cut-off for test positivity. Plasma samples were batched, de-identified, and shipped to the laboratory for testing with the Epi proColon test kit. Data were compiled at the laboratory and the final data set was forwarded to the trial sponsor for analysis following data lock. The data were analyzed internally and corroborated by an external statistics group.

### Statistical Analysis

Sensitivity and specificity estimates and standard 95% confidence intervals (score method) were calculated for all samples tested with either Epi proColon or FIT, regardless of whether there was a valid comparator result. The impact of demographic variables on test performance was analyzed using likelihood ratio tests and significance was determined at P<0.05.

Comparison of performance of Epi proColon and FIT test was based on confidence intervals for paired data for differences of sensitivity and specificity between the two test methods. These measures were used to evaluate non-inferiority of Epi proColon as compared with OC-FIT CHECK. Non-inferiority margins were set to 10% for CRC sensitivity and 20% for specificity.

Sample size (n = 100) for sensitivity estimation was set based on a power calculation that ranged from 70% to 90% depending on the degree of overlap (concordance) between positive calls for both tests in CRC subjects. Test performance of Epi proColon was considered non-inferior to FIT in CRC subjects if the two sided 95% confidence interval for the difference of sensitivities of FIT and Epi proColon was strictly below the non-inferiority margin of 10%.

Sample size (n = 200) for specificity estimation was set based on a power calculation of >90% depending on the degree of overlap (concordance) between negative calls for both tests in non-CRC subjects. The Epi proColon test was considered non-inferior to FIT in non-CRC subjects if the two sided 95% confidence interval for the difference of specificities of FIT and Epi proColon was strictly below the non-inferiority margin of 20%.

## Results

**Figure 1:**
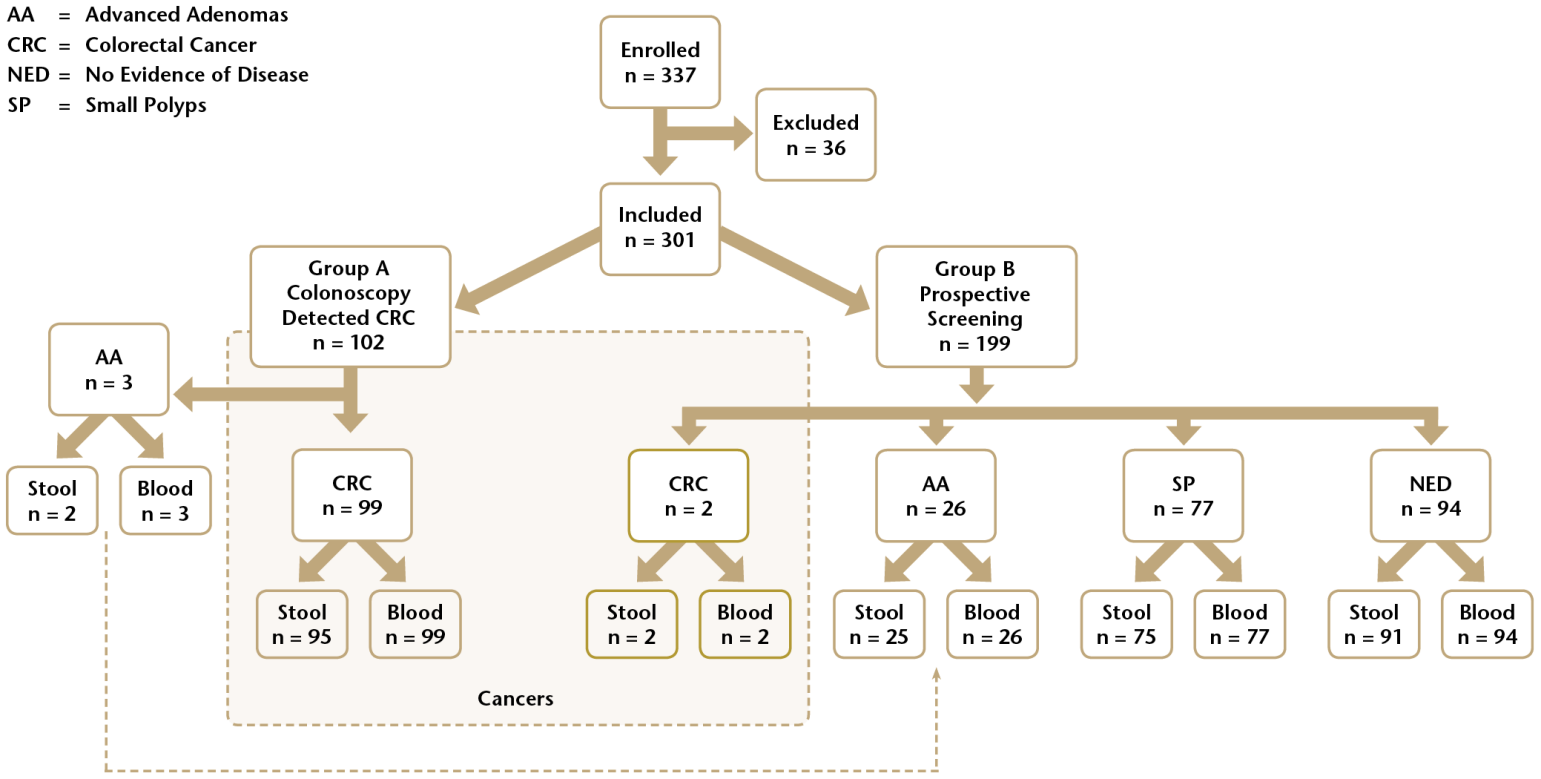
The number of enrolled subjects by class, and the number of blood and stool samples evaluated in the study.

Of 337 subjects enrolled in the study, 36 were excluded due to failure to meet inclusion/exclusion criteria (Figure 1). Of the 102 Group A subjects, 3 were reclassified as AA based on pathology review and 99 subjects had a confirmed diagnosis of CRC. Of the 199 subjects enrolled in Group B, 2 were diagnosed with CRC, 26 were classified as AA, 77 as SP and 94 as NED. In aggregate, there were 101 CRC subjects and 200 non-CRC subjects of which 29 subjects were classified as AA, 77 as SP and 94 as NED. Demographic data are outlined in Table 1. For the 301 subjects, 301 plasma samples and 290 stool samples were evaluable (Figure 1).

**Table 1:** Demographic data for colonoscopy identified CRC subjects and prospectively enrolled screening subjects.

			Clinical Diagnosis of prospectively enrolled patients				
Demographic		CRC Cases	Total	CRC	AA	SP	NED
		n (%)	n (%)	n (%)	n (%)	n (%)	n (%)
Gender	Female	33 (32)	122 (62)	0	14 (11)	47 (39)	61 (50)
	Male	69 (68)	77 (38)	2 (3)	12 (16)	30 (39)	33 (43)
Age	50–59	24 (24)	127 (64)	1 (1)	16 (13)	50 (39)	60 (47)
	60–69	38 (37)	49 (25)	1 (2)	7 (14)	17 (35)	24 (49)
	>69	40 (39)	23 (11)	0	3 (13)	10 (43)	10 (43)
Ethnicity	Black	11 (11)	27 (14)	0	3 (11)	12 (44)	12 (44)
	Caucasian	70 (69)	140 (70)	1 (1)	21 (15)	53 (38)	65 (44)
	Hispanic	17 (17)	24 (12)	1 (4)	2 (8)	5 (21)	16 (67)
	Other	4 (4)	8 (4)	0	0	7 (88)	1 (12)
Total		102 (100)	199 (100)	2 (1)	26 (13)	77 (39)	94 (47)

CRC = colorectal cancer, AA = advanced adenoma, SP = small polyps, NED = no evidence of disease.

The Epi proColon and OC FIT-CHEK sensitivity for CRC were calculated for all measured samples and for paired samples with data for both tests (Table 2). FIT detected 66/97 CRCs or 68.0% (95% CI 58.2–76.5%), and Epi proColon detected 74/101 CRCs or 73.3% (95% CI 63.9–80.9%). For subjects with paired results, FIT detection was identical, and Epi proColon detected 70/97 CRCs or 72.2% (Table 2). Specificity was estimated for all non CRC subjects combined, regardless of classification (AA, SP or NED). The positive rate for FIT was 5/193 (specificity 97.4% (95% CI 94.1–98.9%)) and for Epi proColon was 37/200 (81.5% (95% CI 75.5–86.3%)) (Table 2). Specificity for samples with paired tests was 80.8% and 97.4% for Epi proColon and FIT respectively (Table 2).

**Table 2:** Sensitivity and specificity for all valid FIT and Epi proColon results and for paired results.

OC FIT-CHEK n = 290		95% CI	
Sensitivity	68.0% (66/97)	58.2%	76.5%
Specificity	97.4% (5/193)	94.1%	98.9%
**Epi proColon n = 290 (paired samples)**			
Sensitivity	72.2% (70/97)	62.5%	80.1%
Specificity	80.8% (37/193)	74.7%	85.8%
**Epi proColon n = 301 (all samples)**			
Sensitivity	73.3% (74/101)	63.9%	80.9%
Specificity	81.5% (37/200)	75.5%	86.3%

For paired samples, Epi proColon had 4.25% higher sensitivity and the 95% confidence interval for the difference of OC FIT-CHEK minus Epi proColon (−16.2%; 8.1%) was below the pre-set non-inferiority margin of 10%, indicating that Epi proColon sensitivity was statistically non-inferior to FIT. For specificity the difference between tests was 16.6% in favor of FIT, with a 95% confidence limit (10.6%; 22.9%) indicating a significantly lower specificity of Epi proColon. With respect to the pre-set non-inferiority margin of 20%, statistical non-inferiority for specificity was not met.

Diagnostic likelihood ratios (DLRs) provide another means of comparing FIT and Epi proColon results [26]. The positive DLRs estimated in this study for Epi proColon and FIT were 4.0 and 26.3, respectively, driven by the difference in observed positives in non-CRC samples. The estimated negative DLRs for both test methods were identical (0.33). Since there is a strict mathematical relationship between DLRs and predictive values, the estimated negative predictive value for both test methods was identical (99.8%). The estimated positive predictive values were 2.7% and 15.6% for Epi proColon and FIT (using 0.7% for CRC prevalence). There was overlap in the CRC subjects detected by Epi proColon and OC FIT-CHEK with 50/97 cancers detected by both tests. An additional 20 cancers were detected by Epi proColon that were not detected with FIT, and 16 cancers by OC FIT-CHEK that were not detected by Epi proColon (Table 3). The combined sensitivity in the cohort is 88.7% (86/97). The combined detection for non CRC samples was 21.2% (41/193) for a specificity of 78.8%. Comparison of the degree of overlap in detection of advanced adenomas was not informative as a result of the low OC FIT-CHEK detection rate (Table S7 in File S1).

**Table 3:** Positivity comparison for Epi proColon and OC FIT-CHEK for paired samples.

Colorectal Cancer				Non Colorectal Cancer*			
FIT	Epi proColon		Total	FIT	Epi proColon		Total
	Positive	Negative			Positive	Negative	
Positive	50	16	66	Positive	1	4	5
Negative	20	11	31	Negative	36	152	188
Total	70	27	97	Total	37	156	193

*Includes subjects with advanced adenoma, small polyps, and no evidence of disease.

When comparing test performance in early stage cancer (0, I, II) [27] there was no significant difference (Mc Nemar test, p-value  = 1), with Epi proColon detecting 34/48 (70.8%) of subjects compared with 33/48 (68.8%) detection with OC FIT-CHEK (Table 4). Although there were differences in point estimate for stage III and for stage IV observed, these differences were also not significant (Mc Nemar test, p-values  = 0.29 and 0.14, respectively). Analysis of test performance based on tumor location (left or right colon) showed no difference for either test, with an observed 73.1% (left) vs 75% (right) for Epi proColon (n = 88) and 70.6% (left) vs 69.4% (right) for OC FIT-CHEK (n = 87).

**Table 4:** Valid Epi proColon and FIT results for subjects with CRC.

CRC	Epi proColon	Sensitivity	FIT	Sensitivity
		Epi proColon		FIT
		(95% CI)		(95%CI)
Stage 0	2/2	100%	0/2	0%
		(34.2–100%)		(0–65.8%)
Stage I	16/26	61.5%	17/26	65.4%
		(42.5–77.6%)		(46.2–80.6%)
Stage II	16/20	80.0%	16/20	80.0%
		(58.4–91.9%)		(58.4–91.9%)
Stage III	15/23	65.2%	19/23	82.6%
		(44.9–81.2%)		(62.9–93.0%)
Stage IV	12/13	92.3%	7/12	58.3%
		(66.7–99.6%)		(32.0–80.7%)
Unknown	13/17	76.5%	7/14	50.0%
		(52.7–90.4%)		(26.8–73.2%)
**Total**	74/101	73.3%	66/97	68.0%
		(63.9–80.9%)		(58.2–76.5%)

Sensitivity calculation for all CRC samples.

Data for test positivity based on the demographic variables of age, gender and ethnicity are provided in the supporting information supplement (Tables S1– S6 in File S1. We observed some variation in the positive detected fraction for non-CRC subjects based on age, with the highest fraction for subjects in the 60–69 class. Similarly, we observed variation in performance for both tests amongst ethnic groups. However, the likelihood ratio analysis (Table 5) did not show significant effects based on age, gender and ethnicity (all p-values >0.05). Furthermore, there was no significant effect of factors related to co-morbidities, medication or life style (BMI, smoking, alcohol consumption, physical activity) for either test.

**Table 5:** Analysis of the role of demographic variables on test performance.

Variable	Test	Patient Class	P-value
	Epi proColon	CRC	0.649
Age	OC FIT-CHEK	CRC	0.575
	Epi proColon	Non-CRC	0.200
	OC FIT-CHEK	Non-CRC	0.125
	Epi proColon	CRC	0.390
Gender	OC FIT-CHEK	CRC	0.604
	Epi proColon	Non-CRC	0.582
	OC FIT-CHEK	Non-CRC	0.610
	Epi proColon	CRC	0.571
Ethnicity	OC FIT-CHEK	CRC	0.174
	Epi proColon	Non-CRC	0.696
	OC FIT-CHEK	Non-CRC	0.217

Demographic variables were categorized as indicated and compared using the likelihood ratio test where P-values <0.05 are considered significant.

## Discussion

This prospective, multi-center study compared a novel molecular-based blood test for methylated Septin9 with a conventional FIT for CRC screening. The study was designed and powered to assess the primary endpoint of whether the Epi proColon test was statistically non-inferior to a stool test (OC FIT-CHECK). The study was enriched for CRC subjects and therefore, the head-to-head comparison was not in a pure screening population. However, to obtain CRC subjects closely representative of the screening population, a key enrollment requirement for Group A was that they were identified by screening colonoscopy. This implies that these subjects were of average risk when they presented for CRC screening.

The blood-based test was statistically non-inferior to FIT for the detection of CRC and the observed sensitivities of 73% for methylated Septin9 DNA and 68% for FIT are comparable to measurements for these assays when assessed individually in previous studies [19], [21], [28]. Furthermore, there were no relevant differences in tumor detection with respect to tumor location, age or gender, confirming a previous publication showing similar proximal and distal detection with the Epi proColon test [23]. There were no major ethnic differences in tumor detection rates with the exception of the Hispanic subjects (n = 17) where FIT had an unexpectedly low detection rate (47%).

The observed specificity for FIT and Epi proColon were 98% and 82% respectively. While the FIT performance was on the upper threshold of reported specificity [28] the Epi proColon result was lower than previously reported in a prospective study of a previous version of the test [24], but was similar to that observed in the pivotal trial for the Epi proColon test (unpublished data). The apparent reduction in specificity was confirmed to be true detection of methylated Septin9, reflecting the presence of trace amounts of the target. Thus, we speculate that the increased positivity may be attributed to improved DNA recovery, and PCR sensitivity.

Further analysis of the FIT data, for example by adjusting the hemoglobin threshold, was not done, since the objective was to compare the tests as they would be used in the clinical setting. There was no significant impact of age, gender or ethnicity on specificity. Neither test showed any relevant detection of advanced adenomas or small polyps in this study. While the study was powered to compare performance of the two tests in non-CRC subjects, it was not designed to provide results for the full spectrum of screening eligible patients, which would require large population based studies.

Given that the NPV estimate for the Septin9 test and FIT were essentially identical (99.8%), a negative test result provides similar information on the absence of CRC. On this basis, a negative finding for both tests indicates a very low likelihood of having the disease. Among patients with a positive Septin9 test, the proportion with CRC is increased compared with the untested population. However, the difference in PPV for Septin9 (2.7%) compared with FIT (15.6%), indicates a more effective enrichment of CRC cases in FIT positives. Regardless, it needs to be noted that because of the low prevalence of CRC in general (estimated at 0.7%), the PPV for any CRC screening test is relatively low. For example, even with the exceptional specificity of 97.4% of the FIT test in this study, the PPV was only 15.6%.

Given the non-inferior CRC detection, though at a lower specificity, will the Septin9 blood test be useful for CRC screening? Since the five year survival rate is high for early stage CRC, remaining above 70% even for Stage III, the detection of CRC by screening is medically beneficial. Assuming that the availability of a blood test will increase participation in CRC screening programs, a 70% detection rate with the Septin9 test would significantly reduce mortality due to CRC, as was observed in trials demonstrating changes in mortality, incidence and stage shifting based on gFOBT [29], [30]. A similar benefit has been seen in the use of the PAP smear for cervical cancer screening, where, despite sensitivities of only 50%, the test has drastically lowered the incidence and mortality of this disease [31].

However, the 81.5% specificity of the Septin9 test is a potential concern, since at this rate, the blood test will result in more referrals to colonoscopy compared with FIT. In fact, based on the PPV (2.7%), one cancer will be detected for thirty seven colonoscopies performed, compared with one cancer per one hundred forty three colonoscopies in the population that does not receive the Septin9 test. However, it is also worth noting, that more than one third of subjects with a ‘false positive’ test will have polyps upon subsequent colonoscopy. While colonoscopy is not without risks [32], and while the use of Epi proColon testing, compared to FIT, is expected to result in additional colonoscopies, colonoscopy represents a currently recommended and widely used screening methodology and therefore, Epi proColon would not increase the risk above the standard of care.

For CRC screening, models show that compared with no screening, any of several common strategies (such as yearly FOBT/FIT, periodic colonoscopy, or sigmoidoscopy combined with fecal occult blood testing) reduce CRC mortality by roughly similar magnitudes if screening is adhered to over time [33], [34]. Accordingly, CRC screening guidelines include several acceptable strategies [35], [36]. It has also been reported that offering a choice of tests improves participation [8] and for FIT, that offering a one sample test improves participation over multi-sample tests [37]. In a similar manner, the addition of a blood based test choice may further improve adherence to screening, and potentially urther improve screening outcomes.

Since the objective of this study was a direct comparison of test performance between Epi proColon and FIT, we did not compare patient compliance or assess patient preferences. These parameters will ultimately be major drivers of test acceptance. It is clear that blood-based testing for CRC could have advantages of convenience, safety and patient acceptability compared with existing tests. Reports from patient focus groups [38] and a national telephone survey performed by the Colon Cancer Alliance [39] indicate that many people who currently avoid screening would be willing to take a simple blood test. This new test may help to overcome the barriers that have been reported to keep patients from participating in screening programs [40], [41]. Finally, this study was not designed to address the cost-effectiveness of Epi proColon relative to other screening strategies. Going forward, such analyses are needed to further clarify the potential value of this test for CRC screening.

## Conclusion

The results of this prospective multicenter study support the use of the Epi proColon blood test for CRC screening. The study clearly demonstrates that Epi proColon has similar performance for cancer detection as OC FIT-CHEK, a state-of-the-art FIT test. Based on a comparison of the negative diagnostic likelihood ratios, both tests have similar performance in ruling out cancer in patients with negative test results. The Septin9 test did have a higher positivity for subjects who were negative for CRC, and would result in an increased number of follow-up colonoscopies. However, colonoscopy is a standard of care for CRC screening. The availability of non-invasive screening options, including the Septin9 test based on a simple blood draw, has the potential to increase screening rates and identify additional cancer cases at an early and curable stage.

## Supporting Information

File S1 **This file contains: Table S1:** Sensitivity for Epi proColon and FIT by Age Group. **Table S2:** Positivity in non-CRC subjects for Epi proColon and FIT by Age Group. **Table S3:** Sensitivity for Epi proColon and FIT by Gender. **Table S4:** Positivity in non-CRC subjects for Epi proColon and FIT by Gender. **Table S5:** Sensitivity for Epi proColon and FIT by Ethnicity. **Table S6:** Positivity in non-CRC subjects for Epi proColon and FIT by Ethnicity. **Table S7:** Two way comparison of performance of Epi proColon and OC FIT-CHEK for subjects with advanced adenomas.
(DOCX)

Checklist S1 **TREND Checklist.**
(PDF)

Protocol S1 **Trial Protocol.**
(PDF)
