# Supplementary material for: Plasma Septin9 versus Fecal Immunochemical Testing for Colorectal Cancer Screening: A Prospective Multicenter Study
Source: PLoS One. 2014 Jun 5;9(6):e98238. doi: 10.1371/journal.pone.0098238 (PMC4046970; doi:10.1371/journal.pone.0098238)
Supplement: File S1 — This file contains: Table S1: Sensitivity for Epi proColon and FIT by Age Group. Table S2: Positivity in non-CRC subjects for Epi proColon and FIT by Age Group. Table S3: Sensitivity for Epi proColon and FIT by Gender. Table S4: Positivity in non-CRC subjects for Epi proColon and FIT by Gender. Table S5: Sensitivity for Epi proColon and FIT by Ethnicity. Table S6: Positivity in non-CRC subjects for Epi proColon and FIT by Ethnicity. Table S7: Two way comparison of performance of Epi proColon and OC FIT-CHEK for subjects with advanced adenomas. (DOCX) [file pone.0098238.s001.docx]

Supplemental Table S1: Sensitivity for Epi proColon and FIT by Age Group

| Age group (years) | Epi proColon | Sensitivity  Epi proColon  (95% CI) | FIT | Sensitivity FIT  (95%CI) |
| --- | --- | --- | --- | --- |
| 50 – 59 | 18/25 | 72.0%  (52.4-85.7%) | 18/24 | 75.0%  (55.1-88.0%) |
| 60 – 69 | 29/37 | 78.4%  (62.8-88.6%) | 25/36 | 69.4%  (53.1-82.0%) |
| 70 – 88 | 27/39 | 69.2%  (53.6-81.4%) | 23/37 | 62.2%  (46.1-75.9%) |

Supplemental Table S2: Positivity in non-CRC subjects for Epi proColon and FIT by Age Group

| Age group (years) | | Epi proColon | Positivity  Epi proColon  (95% CI) | FIT | Positivity FIT  (95%CI) |
| --- | --- | --- | --- | --- | --- |
| 50 - 59 | | 19/126 | 15.1%  (9.9-22.4-%) | 1/122 | 0.8%  (0.0-4.5%) |
| 60 - 69 | 14/50 | 28.0%  (17.5-41.7%) | 1/48 | 2.1%  (0.0-10.9%) |  |
| 70 - 88 | | 4/24 | 16.7%  (6.7-35.9%) | 3/23 | 13.0%  (4.5-32.1%) |

Supplemental Table S3: Sensitivity for Epi proColon and FIT by Gender

| Gender | Epi proColon | Sensitivity  Epi proColon  (95% CI) | FIT | Sensitivity FIT  (95%CI) |
| --- | --- | --- | --- | --- |
| Female | 26/33 | 78.8%  (62.2-89.3%) | 19/30 | 63.3%  (45.5-78.1%) |
| Male | 48/68 | 70.6%  (58.9-80.1%) | 47/67 | 70.2%  (58.3-79.8%) |
| Total | 74/101 | 73.3%  (63.9-80.9%) | 66/97 | 68.0%  (58.2-76.5%) |

Supplemental Table S4: Positivity in non-CRC subjects for Epi proColon and FIT by Gender

| Gender | Epi proColon | Positivity  Epi proColon  (95% CI) | FIT | Positivity FIT  (95%CI) |
| --- | --- | --- | --- | --- |
| Female | 24/122 | 19.7%  (13.6-27.6%) | 3/120 | 2.5%  (0.1-7.1%) |
| Male | 13/78 | 16.7%  (10.0-26.5%) | 2/73 | 2.7%  (0.8-9.5%) |
| Total | 37/200 | 18.5%  (13.7-24.5%) | 5/193 | 2.6%  (1.1-5.9%) |

Supplemental Table S5: Sensitivity for Epi proColon and FIT by Ethnicity

| Ethnicity | Epi proColon | Sensitivity  Epi proColon  (95% CI) | FIT | Sensitivity FIT  (95%CI) |
| --- | --- | --- | --- | --- |
| African-American | 9/10 | 90.0%  (59.6-99.5%) | 7/10 | 70.0%  (39.7-89.2%) |
| Caucasian | 50/70 | 71.4%  (60.0-80.7%) | 50/68 | 73.5%  (62.0-82.6%) |
| Hispanic | 12/17 | 70.6%  (46.9-86.7%) | 7/15 | 46.7% (24.8-69.9%) |
| Other | 3/4 | 75.0% (30.1-98.7) | 2/4 | 50.0% (15.0-85.0%) |
| Total | 74/101 | 73.3%  (63.9-80.9%) | 66/97 | 68.0%  (58.2-76.5%) |

Supplemental Table S6: Positivity in non-CRC subjects for Epi proColon and FIT by Ethnicity

| Ethnicity | Epi proColon | Positivity  Epi proColon  (95% CI) | FIT | Positivity FIT  (95%CI) |
| --- | --- | --- | --- | --- |
| African-American | 6/28 | 21.4%  (10.2-39.5%) | 0/27 | 0.0%  (0.0-12.5%) |
| Caucasian | 24/140 | 17.1%  (11.8-24.2%) | 2/134 | 1.5%  (0.4-5.3%) |
| Hispanic | 6/24 | 25.0% (12.0-44.9%) | 3/24 | 12.5% (4.3-31.0%) |
| Other | 1/8 | 12.5% (0.6-47.1%) | 0/8 | 0.0% (0.0-3.2%) |
| Total | 37/200 | 18.5%  (13.7-24.5%) | 5/193 | 2.6%  (1.1-5.9%) |

Supplemental Table S7: Two way comparison of performance of Epi proColon and OC FIT-CHEK for subjects with advanced adenomas

| FIT | Epi proColon | | Total |
| --- | --- | --- | --- |
|  | Positive | Negative |  |
| Positive | 1 | 1 | 2 |
| Negative | 3 | 22 | 25 |
| Total | 4 | 23 | 27 |
