# Supplementary material for: Plasma Septin9 versus Fecal Immunochemical Testing for Colorectal Cancer Screening: A Prospective Multicenter Study
Source: PLoS One. 2014 Jun 5;9(6):e98238. doi: 10.1371/journal.pone.0098238 (PMC4046970; doi:10.1371/journal.pone.0098238)
Supplement: Protocol S1 — Trial Protocol. (PDF) [file pone.0098238.s003.pdf]

Document Title: Comparison of the Performance of the Epi proColon Test and Fecal Immunochemical Testing (FIT) post colonoscopy in Subjects with Colorectal Cancer (CRC) and pre colonoscopy in Subjects

Document Number: SPR 0022P

Revision Level	Effective Date	DCN	Description of Revision	Revision Author
Rev 1	29/12/2011	3580	Initial release.	Neil Mucci

Approval:

Department (name signatory)	Signature	Date (dd.mm.yy)
Project Manager (Gunter Weiss)		
Study Director/Medical Affairs (Jürgen Beck)		
Marketing (Philipp Schatz)		
Clinical Research (Kerstin Buser)		
Study Management (Neil Mucci)		
Biostatistics (Thomas Koenig)		
Regulatory (Catherine Lofton-Day)		
Quality Assurance (Elli Neu)		

Contents

1.0 General Information **4**
1.1 Protocol Title 4
1.2 Short Title 4
1.3 Protocol Number 4
1.4 Name and Address of the Sponsor 4
1.5 Name and Title of the Person(s) Authorized to Sign Protocol and Protocol Amendments for the Sponsor 4
1.6 Name and Title of the Sponsor's Medical Expert for the Investigation 4
1.7 Name and Title of the Monitors 4
2.0 Background Information **4**
2.1 Rationale for the Study 4
2.2 Technical Data to Date 4
2.3 Study Summary 5
3.0 Trial Objectives and Purpose **5**
3.1 Primary Objective of the Study 5
4.0 Trial design **5**
4.1 General Design 5
4.2 Investigational Plan 5
4.3 Sample Number and Amounts 6
4.4 Randomization and Blinding 6
5.0 Selection and Withdrawal of Subjects **6**
5.1 Inclusion Criteria 6
5.2 Exclusion Criteria 7
6.0 Treatment of Subjects **7**
6.1 Blood Specimen and Sampling Requirements 7
6.2 Blood Collection (at the investigational site) 7
6.3 Stool Specimen and Sampling Requirements 8
6.4 Stool Samples collection 8
6.5 Plasma Specimen Preparation (at the laboratory) 8
6.5.1 Blood Specimen Inclusion Criteria 9
6.5.2 Blood Specimen Exclusion Criteria 9
6.6 Treatments authorized/not permitted before and/or during the specimen collection 9
6.7 Treatment of Plasma Specimens at Epigenomics or at an External Laboratory 9
6.8 C-FIT CHEK Testing Procedures 10
7.0 Assessment of Efficacy **10**
8.0 Assessment of Safety **10**
9.0 Statistical Analysis **10**
9.1 Reporting Results, Descriptive Measures 10
9.2 Statistical Inference, Sample Size 11
9.3 Hypothesis Acceptance / Validity Criteria 13
9.3.1 Sensitivity for colorectal cancer 13
9.3.2 Specificity for subject without evidence of disease 13

9.4 Additional Analyses 13
9.5 Procedure for account for missing, data 13
9.6 Selection of subjects to be included in the analysis 13
10.0 Direct Access to Source Data /Documents 13
10.1 Case Report Forms 13
10.2 Source Documents 14
10.3 Direct Access to Source Data / Documents 14
11.0 Quality Control and Quality Assurance 14
11.1 Training 14
11.2 Study Monitoring Plan 15
11.2.1 The main goals of the monitoring visits are: 15
11.2.2 Monitoring Visit Activities: 15
11.3 Incoming Quality Control at Epigenomics or designee 15
11.4 Auditing and Inspecting 17
11.5 Ethical Considerations 17
11.6 Conflict of Interest 18
12.0 Data Handling and Record Keeping 18
12.1 Confidentiality 18
12.2 Case Report Forms 18
12.3 Records Retention 18
12.4 CRF/ Essential Record Storage and Archiving 19
12.5 Other Study Related Forms 19
12.5.1 Delegation of Authority 19
12.5.2 Contact Information Form 20
12.6 Deviations from the Protocol 20
12.7 Changes to the Protocol 20
12.8 Study Report 20
12.9 Software for Data Collection 20
13.0 Financing and Insurance 21
14.0 Publication Plan 21
15.0 Supplements 21
16.0 References 22

1.0 General Information

1.1 Protocol Title

Comparison of the performance of the Epi proColon - test and Fecal Immunochemical Testing (FIT) post colonoscopy in subjects with Colorectal Cancer (CRC) and pre colonoscopy in subjects from guideline-eligible screening population.

1.2 Short Title

Head to Head Study Epi proColon and FIT

1.3 Protocol Number

SPR 00XX

1.4 Name and Address of the Sponsor

Epigenomics AG, Kleine Präsidentenstraße 1, 10178 Berlin, Germany with its fully owned subsidiary Epigenomics Inc., 901 5th Avenue, Suite 3800 Seattle, Washington 98164 U.S.A.

1.5 Name and Title of the Person(s) Authorized to Sign Protocol and Protocol Amendments for the Sponsor

Same as approver list on front page.

1.6 Name and Title of the Sponsor's Medical Expert for the Investigation

Dr. Jürgen Beck, Senior Vice President Medical Affairs

1.7 Name and Title of the Monitors

In addition to the above named Sponsor Representatives, other authorized and GCP trained personnel from Epigenomics or designee will act as Study Monitors.

2.0 Background Information

2.1 Rationale for the Study

The study will be conducted with the aim to show non-inferiority in the clinical performance of the investigational Epi proColon assay to FIT - OC FIT-CHEK® (Polymedco) - using matched blood and stool specimens from screening-guideline eligible subjects.

2.2 Technical Data to Date

Epigenomics has identified methylated gene regions that are specific for colorectal cancer (CRC)[1]. Through Epigenomics' marker discovery and validation process, DNA methylation of a region of the Septin9 gene was identified as a robust biomarker for detection of CRC[2]. Epigenomics developed a blood test for CRC based on the detection of the Septin9 biomarker called Epi *pro*Colon Early Detection Assay, available as a CE-

marked kit in Europe since October 2009. According to the results of the performance evaluation study[3], the first generation product detected approximately 70 % of the CRC cases with a specificity close to 90 %. In a CRC screening cohort (PRESEPT[4]), the Septin9 biomarker assay detected 67 % of CRC cases with a specificity of 89 %. A second generation Septin9 biomarker assay with improved clinical performance characteristics has been developed. Epigenomics is performing the current study to directly compare the second generation Epi *pro*Colon 2.0 assay with the current non-invasive standard for colorectal screening, FIT, in order to provide supporting data for safety and effectiveness of the Epi proColon 2.0 investigational device for its proposed intended use.

2.3 Study Summary

This study is designed to prospectively collect matched blood and stool specimens and clinical data from screening guideline-eligible subjects found to have invasive colorectal cancer (CRC) at colonoscopy, i.e. AJCC/UICC stages I, II, III, and IV, with collection of specimens and testing after colonoscopy and from screening guideline-eligible subjects with blood and stool specimens collected before colonoscopy.

3.0 Trial Objectives and Purpose

3.1 Primary Objective of the Study

The primary objective of the investigation is to show that the clinical performance in terms of test positivities of the investigational Epi proColon test is non-inferior to OC FIT-CHEK®, (Polymedco), a commonly used and commercially available (in the United States) fecal immunochemical test (FIT) used for the detection of colorectal cancer.

4.0 Trial design

4.1 General Design

The study is designed as a prospective case-control clinical investigation involving up to 40 active study sites in the United States and in Europe. European enrollment will be limited to 20% of the subjects. Subjects interested in participation and who provide written informed consent will be enrolled in the study provided inclusion criteria are met. Eligible, consented CRC subjects and controls will have up to 40 ml of blood drawn and will provide the amount of stool required per manufacturer’s instructions of the OC FIT-CHEK® test. DNA obtained from plasma will be bisulfite-converted and assessed for presence of methylated Septin 9 following the instructions for use provided with the investigational Epi proColon test. Stool samples will be processed in accordance with the manufacturer’s instructions.

4.2 Investigational Plan

The clinical performance of the investigational Epi proColon test will be compared to that of OC FIT-CHEK®.

4.3 Sample Number and Amounts

This study aims to enroll two different subsets of subjects which are described below. In order to include into the study the following numbers of fully compliant subjects, the estimate is that we need to recruit 200 subjects per group (assumes 50% attrition rate, therefore 100 evaluable per group.)

Group A: 100 subjects between age 50 and 84 having CRC, consented and specimens collected and tested after colonoscopy

Group B: 100 subjects between age 50 and 84, consented and tested before colonoscopy and bowel preparation. It is planned to obtain both blood and stool samples from all subjects enrolled into the study. Both Group A and B must include 100 subjects each who have provided both a blood sample and stool sample for FIT. The study will be continued until these quotas will have been reached. Enrolled subjects not providing both samples according to this protocol will be considered screen failures and will not be evaluated.

Enrollment will be conducted so that ages of subjects in the two groups are not disparate. These sample numbers are considered appropriate for the calculations mentioned in the following paragraph.

4.4 Randomization and Blinding

Randomization will not occur: Only subjects who provide both types of samples will be evaluated. The laboratories performing the tests will be blinded with regard to the subject’s clinical diagnosis.

5.0 Selection and Withdrawal of Subjects

Group A CRC subjects participating in the study will be selected and sourced from an average-risk, screening guideline eligible population who have undergone colonoscopy and who have been diagnosed with colorectal cancer. Group B subjects will be enrolled from an average-risk, symptom-free population who are scheduled to have colonoscopy.

5.1 Inclusion Criteria

Group A

1) Willing and able to sign an IC and adhere to study requirements.
2) Both genders eligible.
3) 50 – 84 years of age at blood & stool sampling.
4) Colonoscopic diagnosis of colorectal carcinoma (CRC).*
5) Colonoscopy within 6 months before inclusion into the study, i.e. latest colonoscopy must be in the past 6 months.
6) Blood & stool sampling a minimum of 10 days after colonoscopy and before resection surgery.

** = strong clinical suspicion of colorectal carcinoma is also acceptable for subject enrollment; Case must have a confirmed diagnosis after surgery of CRC and be accompanied by a complete final pathology report.*

Group B

1) Willing and able to sign an IC and adhere to study requirements.
2) Both genders eligible.
3) 50 – 84 years of age at blood and stool sampling.

5.2 Exclusion Criteria

Group A Only:

1) Subject with curative biopsy during colonoscopy.

Group A and B:

1) Previous personal history of CRC or previous colonoscopy resulting in a recommendation to repeat colonoscopy at an interval less than ten years (high risk population).
2) Neoadjuvant treatment
3) Familial risk for colorectal cancer (2 or more 1st degree relatives with CRC; 1 or more 1st degree relative(s) < 50 years with CRC; known HNPCC or FAP).
4) History of inflammatory bowel disease.
5) Acute or chronic gastritis.
6) Current diagnosis of any other cancer than CRC.
7) Overt rectal bleeding or bleeding hemorrhoids.
8) Known infection with HIV, HBV or HCV.
9) Subject concurrently receiving intravenous fluid at the time of the specimen collection.

6.0 Treatment of Subjects

6.1 Blood Specimen and Sampling Requirements

Plasma specimens will be prepared according to the Instructions for Use of the Epi proColon investigational device (IFU 0008) with the exception that multiple blood tubes will be drawn and multiple aliquots of plasma prepared for each subject. Consented subjects will provide up to 40 ml of blood.

6.2 Blood Collection (at the investigational site)

Vacutainer® EDTA Tube (Becton Dickinson, catalogue no. 366643) 10 ml tube will be used for drawing blood. Whole blood samples must not be frozen.

Processing of whole blood must begin within four hours of blood collection. After initial collection of plasma from centrifuged whole blood or after a second centrifugation of collected plasma, specimen can be stored at 2 to 8°C for up to 72 hours prior to further processing or freezing.

6.3 Stool Specimen and Sampling Requirements

Stool samples for FIT will be collected and tested according to the manufacturer's instructions for use for OC FIT-CHEK® (PolyMedco).

6.4 Stool Samples collection

The subjects will be instructed, according to manufacturer's instructions, to collect the stool sample as follows:

1) place the supplied collection paper inside toilet bowel on top of water,
2) deposit stool sample on top of collection paper,
3) collect test sample from stool sample before paper sinks and stool sample touches water,
4) fill in required information (name, age, gender, date) on sampling bottle,
5) open sampling bottle,
6) scrape the surface of the fecal sample with the sample probe,
7) cover the grooved portion of the sample probe completely with stool sample,
8) close sampling bottle by inserting the sample probe and snap cap on tightly,
9) do not reopen sampling bottle,
10) keep sampling bottle between 2 and 30°C,
11) put the sample in the envelope and return it to the laboratory the same day.

The study nurse will follow-up with the subject by phone to remind her/him to provide the stool samples.

6.5 Plasma Specimen Preparation (at the laboratory)

- Disable the brake function in the centrifuge to prevent disruption of the cell layer.
- Centrifuge the blood in Vacutainer® (or S-Monovette tubes) for 12 min at 1350 ± 150 rcf. If centrifuge uses RPM (revolutions per minute), refer to the centrifuge user manual for a conversion table.
- Remove Vacutainer® from centrifuge. Discard the sample if haemolysis (bright red plasma) is observed. In this case a new blood sample must be taken.

- Use a Pasteur single-use pipette to transfer plasma from the collection tube to a 15 ml polypropylene centrifuge tube with conical bottom.
- Centrifuge plasma in the 15 ml centrifuge tube for 12 min at 1350 ± 150 rcf.
- Using a new bulb pipette, transfer 4.0 ml plasma into labelled cryovials or centrifuge tubes.

6.5.1 Blood Specimen Inclusion Criteria

- At least 12 ml plasma sample per patient.
- Plasma collected, processed, shipped and stored according to provided Instructions for Specimen Processing.
- All CRFs complete including complete staging information for colorectal cancer cases.

6.5.2 Blood Specimen Exclusion Criteria

- Specimen thawed after initial freezing (except at testing).
- Gross hemolysis of plasma specimen.

6.6 Treatments authorized/not permitted before and/or during the specimen collection

Processed blood specimens will be stored at the site or the Laboratory, which processed the plasma until bulk shipment as requested by the Sponsor. Packaging of blood specimens and shipping will be performed according to Specimen Shipment Instructions using a courier specialized in shipping frozen specimen for diagnostic use. Temperature may be monitored during shipment with single use temperature monitoring devices provided by the Sponsor indicating a temperature above -1 °C.

Specimens will be stored in a –70 °C (or colder) freezer at Epigenomics or designee until they are analyzed by Epigenomics or shipped to authorized laboratories.

6.7 Treatment of Plasma Specimens at Epigenomics or at an External Laboratory

Upon arrival of the specimens at Epigenomics’ or designee’s facilities, Epigenomics or designee will perform the following quality control on the specimens:

- Specimen is frozen upon receipt
- Accurate identification and labeling of specimen tubes
- Minimum of 12 ml plasma volume
- No evidence of gross hemolysis or turbidity

Only plasma specimens that have passed this quality control will be included in the analysis. The result of these quality controls will be documented at Epigenomics or designee during the check-in process, and communicated to the Provider.

All experiments may be conducted in cooperation with commercial partners of Epigenomics either in the facilities of Epigenomics or at the facilities of the respective authorized partner. Data will be shared with Epigenomics' partners as it relates to the study herein defined.

Surplus sample material will remain at Epigenomics or designee. Other molecular analysis may be performed whether or not contemplated by this Study Protocol. No genetic profiling is foreseen in the context of the investigation.

6.8 C-FIT CHEK Testing Procedures

Testing will take place in the central laboratories (one in the US, one in Europe) using the manufacturer approved automated systems OC-Auto Micro 80 or OC-Sensor Diana at an 100 ng/ml cut-off, The central laboratories will also provide the raw QUANTITATIVE data on request for further analysis. The laboratory doing the testing will report the result directly into the specific section of the CRF without having access to other information contained in the CRF. There are no medicinal or dietary restrictions for the use of OC-FITCHEK.

7.0 Assessment of Efficacy

The FIT and Epi proColon assessments will be compared to medical diagnosis/colonoscopy for determination of sensitivity and specificity and reported along with 95% confidence intervals for the point estimates. Positive and negative percent agreement between FIT assessment and Epi proColon assessments will also be provided with corresponding 95% confidence intervals.

8.0 Assessment of Safety

Adverse Events will be reported, if applicable.

9.0 Statistical Analysis

9.1 Reporting Results, Descriptive Measures

A completed subject will have a FIT assessment, an Epi proColon assessment, and a medical diagnosis/colonoscopy determined CRC/NED status. Demographic and baseline covariates will be reported for each individual. Medical diagnosis/colonoscopy is regarded as a reference standard. FIT is regarded as a non-reference standard.

For Group A (CRC) all completed individuals with valid results will be reported in tabular form. The number of individuals considered disease positive and disease negative will be reported as determined by FIT and Epi proColon. Screen failures and incomplete cases will be provided in listings. From the tabular results the proportion (or percent) regarded as disease positive will be calculated for both FIT and Epi proColon. In both cases the numerator will be the number of test positives identified correctly by each test

(confirmed by medical diagnosis/colonoscopy) and the denominator will be the number identified by medical diagnosis/colonoscopy as disease positive. Additionally the proportion (or percent) agreement will be calculated between FIT and Epi proColon and regarded as the ‘positive percent agreement’ and 95% confidence intervals will be provided.

For Groups B (NED) all completed individuals with valid results will be reported in tabular form. For each group the total number of NED individuals will be reported as determined by medical diagnosis/colonoscopy for complete subjects. In tabular form the number of individuals considered disease negative and disease positive will be reported as determined by FIT and Epi proColon. Screen failures and incomplete cases will be provided in listings.

From the tabular results the proportion (or percent) regarded as disease negative will be calculated for both FIT and Epi proColon. In both cases the numerator will be the number of test negatives identified correctly by each test (confirmed by medical diagnosis/colonoscopy) and the denominator will be the number of test negatives identified by medical diagnosis/colonoscopy. Additionally the proportion (or percent agreement will be calculated between FIT and Epi proColon and regard as the ‘negative percent agreement’ and 95% confidence intervals will be provided.

Sensitivities and specificities will be calculated in the above cases where FIT and Epi proColon will be compared to medical diagnosis/colonoscopy as reference standard. Parameters estimated and 95% confidence intervals will be provided.

Listing or line data will be provided for all studies which will include a complete listing of samples with demographic information, clinical diagnosis and the results of both assays. False negatives, false positives, and nonconcordance between FIT and Epi proColon will be described in detail.

Details of data displays in tabular, listing and figure format will be detailed in the Statistical Analysis Plan as recommended in the FDA Guidance ‘Design Considerations for Pivotal Clinical Investigations for Medical Devices’.

9.2 Statistical Inference, Sample Size

In addition to the descriptive methods described in the previous section, statistical inference will be based on sensitivity and specificity as well as positive percent agreement and negative percent agreement.[6]

Clinical performance of Epi pro Colon and FIT will be compared in terms of:

- Sensitivity for colorectal cancer (CRC) relative to reference standard.
- Specificity for no evidence of disease (NED) relative to reference standard.

The sensitivities of Epi proColon and FIT will be compared to establish non-inferiority. Likewise the specificities of Epi proColon and FIT will be compared to establish equivalence.

Non-inferiority of the Epi proColon assessment when compared to FIT assessment will be evaluated using a McNemar's test with type I error rate 0.05. For both sensitivity and specificity differences 95% confidence intervals will be provided.

For the sample size calculation, the primary comparison of sensitivities and specificities is considered to be an non inferiority test for two correlated proportions.
With a type I error rate of 5% and 80% power a sample size of 100 subjects per Group would be required. The maximum allowable difference between the sensitivities (Epi proColon compared to FIT) and specificities (Epi proColon compared to FIT) that still results in non-inferiority (non-inferiority margin) is 0.20.

An attrition rate of 50% wil be assumed. Therefore, in order to collect 100 valid samples per group, a minimum of 200 subjects will be enrolled per group.

9.3 Hypothesis Acceptance / Validity Criteria

9.3.1 Sensitivity for colorectal cancer

Test performance of the Epi proColon test is considered non-inferior to FIT in CRC subjects, if the sensitivities of Epi proColon and FIT (both relative to reference standard medical diagnosis/colonoscopy) are determined to be equivalent by showing a difference no greater than 0.20 in the two sensitivities.

9.3.2 Specificity for subject without evidence of disease

Test performance of the Epi proColon test is considered non-inferior to FIT in NED subjects, if the specificities of Epi proColon and FIT (both relative to reference standard medical diagnosis/colonoscopy) are determined to be equivalent by showing a difference no greater than 0.20 in the two specificities.

9.4 Additional Analyses

Subgroup analysis will be conducted on age, race, and clinical site provided reportable numbers are available and other demographic factors collected on the case report forms. Tabular presentations will be provided showing frequency distributions and line listings will be provided separately.

Center descriptive statistics will be presented to show the effect of center.
As a secondary analysis, the primary analysis will also be conducted as a superiority hypothesis test.

In addition, sensitivity and specificity will be calculated for all samples tested with Epi proColon, reagrdless of whether or not there is a valid FIT result.

9.5 Procedure for account for missing, data

Imputation for incomplete cases will not be employed since recruitment will be continued when screen failures occur to assure the sample numbers are achieved. Cases considered to be invalid test results for any reason will not be used in the analysis but will be provided in data listings.

9.6 Selection of subjects to be included in the analysis

All valid samples obtained from consented patients will be used in the analysis.

10.0 Direct Access to Source Data /Documents

10.1 Case Report Forms

Case Report Forms (CRFs) should be filled in by the Principal Investigator for the institution, or his/her designee. The CRFs will contain the clinical information obtained

from the subject clinical record and the information for the specimen processing at the laboratory(s) where the blood collection and plasma processing is done.

The CRFs will contain the following information per case:

- Patient Demographics : Gender, age at blood draw, height, weight, ethnicity
- Unique subject (and specimen) code
- Clinical inclusion criteria
- Clinical exclusion criteria
- Colonoscopy information and associated histological data when applicable
- Pathological information from the biopsy/surgical specimen when applicable
- Presence of co-morbidities and exposure to medication
- Tracking information for specimen collection and processing
- Lifestyle information:
    - Current smoking information (type of use, number smoked by day, number years of use)
    - Past smoking information (type of use, number smoked by day, number years of use)
    - Alcohol information (average drinks per day)
    - Diet information (diet type)
    - Exercise information (frequency of exercise, average duration of exercise per day, type of exercise)

10.2 Source Documents

For this investigation, the source documentation will be the subject's clinical record including colonoscopy and pathology report, and the enrollment log.

10.3 Direct Access to Source Data / Documents

Epigenomics' clinical monitors and auditors, IRB/IEC representatives and, if applicable, FDA inspectors or representatives from other regulatory agencies, as applicable, must have access to all source documents as required by the protocol.

11.0 Quality Control and Quality Assurance

11.1 Training

The Principal Investigator and other study personnel will receive training on the protocol from the Sponsor or its designated CRO personnel before the study starts. Training sessions will be documented on the direct training form and a copy of the form will be provided to the Sponsor, or CRO.

11.2 Study Monitoring Plan

The study will be monitored according to a study monitoring plan. The Principal Investigator will allocate adequate time for such monitoring activities. The Principal investigator will also ensure that the monitor or other compliance or quality assurance reviewer is given access to all study and study–related documents and underlying source documentation required by this protocol and study related facilities including diagnostic laboratories and has adequate space to conduct the monitoring visit.

Monitoring visits will be scheduled with the site PI and other investigation-related personnel for the site such that there is at least one visit during the specimen and data collection and one after data and specimen collection has finished (this might be done together with the Site Close Out Visit). The monitoring visits will be scheduled for mutually convenient times.

11.2.1 The main goals of the monitoring visits are:

- Assure compliance with all aspects of the provided protocol.
- Assure compliance with the informed consent process, if applicable.
- Ensure that the specimen collection instructions are properly followed.
- Ensure data quality, such as accuracy, correctness, legibility and completeness.
- Check that all required documentation is present and complete in the site's Regulatory Binder.
- Discuss with the PI, or investigation-related personnel any problems that may occur during the collection which were not foreseen at the Site Initiation Visit. Whenever possible, solutions should be decided during the visit or a plan on how the problem will be solved should be agreed upon.

11.2.2 Monitoring Visit Activities:

In order to fulfill the aforementioned goals, each monitoring visit may consist of the following, depending on the time-point of the visit and the needs of the particular site and/or PI:

- Review of given informed consents.
- Source data check and data verification.
- Meeting with the PI appointed for the investigation to monitor the subject enrollment and collection procedures as well as the documentation process.
- Checking the site's Regulatory Binder for completeness.
- Checking storage facilities for the site's Regulatory Binder and specimen collection kits for adequacy.

11.3 Incoming Quality Control at Epigenomics or designee

Specimens need to be collected as described in this protocol. Upon arrival of the specimens at Epigenomics' or designee's facilities, Epigenomics or designee will perform the following quality control on the specimens:

- Visual inspection of the sample tubes' content against the shipment list to verify that shipment and shipment list are identical and that tubes are filled.
- A minimum of 12 ml plasma per subject.

Only specimens that have passed the quality control will be included in the study, and reimbursed. After quality control, specimens will be stored at Epigenomics' facilities or designee.

11.4 Auditing and Inspecting

The investigator(s)/institution(s) will permit study-related monitoring, audits and inspections by the Independent Ethics Committee (IEC) and/or Institutional Review Board (IRB), the sponsor or designee, government regulatory bodies and institution/university compliance and quality assurance groups of all study related documents (e.g. source documents, regulatory documents, data collection instruments, study data etc.). The investigator(s)/ institution(s) will ensure the capability for inspections of applicable study-related facilities such as diagnostic laboratories.

11.5 Ethical Considerations

This study is to be conducted according to applicable international standards of Good Clinical Practice government regulations and Institutional research policies and procedures including FDA Title 21 part 50 (Protection of Human Subjects) and International Conference on Harmonization Guidelines.

This protocol and any amendments will be submitted to a properly constituted Independent Ethics Committee (IEC) or Institutional Review Board (IRB), in agreement with local legal prescriptions, for formal approval of the study conduct. The decision of the IEC/IRB concerning the conduct of the study will be made in writing to the investigator(s)/institution(s) and a copy of this decision will be provided to the sponsor before commencement of this study. The investigator(s)/institution(s) should provide a list of IEC/IRB members and their affiliate to the sponsor.

All subjects will be assigned a unique identifier, (Subject Code) provided by Epigenomics and the logs linking the identifiers and the clinical record number will be kept in the site's Regulatory Binder for the clinical investigation. These logs will allow proper monitoring of the clinical records and/or pathology reports by the appointed clinical monitors in order to assure completeness, accuracy and correctness of the data collected at each site. This unique identifier will also be link to the collected specimens and will be used for all subsequent steps in the clinical investigations.

In order to protect subjects' privacy **none** of the following personal identifiers will be included in any material sent to Epigenomics (adapted from HIPAA identifiers list):

- Names
- Geographic subdivisions (smaller than the state)
- Phone, fax numbers
- Subject's addresses
- Social Security, medical record numbers
- Any other unique number, characteristic or code

Informed consent will be obtained before sample and data collection from each subject. Subjects will only be enrolled in this study if they are able to give informed consent.

Financing and Insurance:

Financing, investigator(s)/institution(s) payment(s) and insurance aspects of the investigation are addressed in the Clinical Investigation Agreement.

11.6 Conflict of Interest

Any investigator who has a conflict of interest with this study (patent ownership, royalties or financial gain greater than the maximum allowable by their institution, etc.) must have the conflict reviewed by a properly constituted Conflict of Interest Committee with a Committee-sanctioned conflict management plan that has been reviewed and approved by the study sponsor prior to participation in this study.

12.0 Data Handling and Record Keeping

12.1 Confidentiality

Individual subjects' information collected for the clinical investigation will be kept confidential at the sample collection sites and/or the participating clinical laboratories (contractually bound to confidentiality with the Sponsor) in charge of performing the investigational testing. Data collected on the CRFs will be entered into a validated database at the Sponsor or designee with limited access.

In order to protect subjects' privacy no personal identifiers will be included in the CRFs.

Information may be disclosed to the FDA and any other regulatory authorities as required to obtain approval or clearance for marketing the product according to national and international regulations.

12.2 Case Report Forms

Paper CRFs or remote electronic data capture (EDC) will be the primary data collection instrument(s) for the study. All data requested must be correct, accurate, legible and complete. CRFs and EDC systems should be filled in by the Principal Investigator for the institution, or his/her designee. Any change or correction should be dated, initialed (signed), and explained (if necessary) by the person responsible for making the change.

Changes to paper records should not obscure the original entry (i.e. an audit trail is maintained). The correct way to make a correction is to cross out the original entry with a single line. No correction fluid may be used. This allows the original entry to remain legible. Example: "cold" sinus infection. The wrong data entry "cold" is crossed out and the correct response, "sinus infection" is written beside the entry. Clearly initial and date beside the lined out item.

12.3 Records Retention

The Principal Investigator(s)/institution(s) is/are responsible for retaining all study documents, including the underlying source documents, according to the regulatory authorities' requirements and guidelines. The PI is responsible for retaining essential

study documents, for the lifetime of the product according to ISO 13485:2003 Medical devices – Quality management systems – Requirements for regulatory purposes.

The PI or Epigenomics may withdraw from the responsibility to maintain records for the period required above and transfer custody of the records to any other person who will accept responsibility for them.

As applicable, a notice of the transfer shall be given to the responsible Regulatory Authorities no later than 10 working days after transfer occurs.

These documents should be retained for a longer period if required by an agreement with Epigenomics. In such an instance, it is the responsibility of Epigenomics to inform the PI as to when these documents are no longer required.

12.4 CRF/ Essential Record Storage and Archiving

Essential records should be maintained in a legible condition. Adequate and suitable space should be provided for the secure storage of all essential records upon the completion of the investigation. The facilities must be secure, with appropriate environmental controls and adequate protection from fire, flood and unauthorized access.

The PI will make Epigenomics aware of the storage arrangements for the documents to be stored at investigator sites. If the investigator becomes unable to store their essential documents, the sponsor should be notified in writing so that alternative storage arrangements can be agreed.

If the institution/site is no longer able to maintain custody of their essential documents, he/she must notify Epigenomics in writing and the investigator needs to ensure that appropriate arrangements can be made.

12.5 Other Study Related Forms

12.5.1 Delegation of Authority

The PI may delegate any of the study-related tasks to other Sub-Investigators or site personnel. The PI will complete a Delegation of Authority Documentation Log to indicate his/her role for each clinical investigation. Activities are officially deemed as delegated once the Principal Investigator signs the Delegation of Authority Documentation Log.

The PI, or designee, is responsible for signing and updating the Delegation of Authority Documentation Log as changes occur until the end of the clinical investigation. The PI, or designee, will store the signed, completed Delegation of Authority Documentation Log in the site's Regulatory Binder, and will send a copy to the Sponsor.

12.5.2 Contact Information Form

The contact information form for the investigation contains names and contact details for the PI and all other site personnel involved in the clinical investigation.

The PI, or designee, is responsible for filling in and updating the contact information form whenever new information is obtained and for storing it in the Regulatory Binder. Any updates to the list must be promptly made available to Sponsor.

12.6 Deviations from the Protocol

All deviations from the protocol related to sample and/or data collection and handling, or deviations from the eligibility, inclusion, exclusion criteria from patients and/or samples which happen during the course of a clinical investigation will be documented.

All deviations must be reported to Epigenomics in a timely manner. Deviations that compromise the rights and welfare of participants or repeated actions or omissions indicating a deficiency in the ability or willingness to comply with regulations will be reported to the IRB/IEC Deviations based on logistical changes or administrative aspects do not require reporting to the IRB/IEC. When a deviation from the study protocol occurs, the following needs to be done:

- Deviations from the study protocol will be logged in a Study Protocol Deviation Log by the PI, or designee. The deviation log will be stored in the Regulatory Binder.
- Deviations will be described in detail in a Study Protocol Deviation Report, a copy sent to Epigenomics and stored together with the subjects' CRFs in the Regulatory Binder.

12.7 Changes to the Protocol

Changes to the protocol, required by Epigenomics, such as changes in the patients' eligibility, inclusion, exclusion criteria or major changes to the sample collection or collection of additional data must be reported to the IRB/IEC.

12.8 Study Report

Epigenomics is responsible for the generation of the Study Report(s). The Study Reports are the final documentation of the results and interpretation of the clinical investigation.

12.9 Software for Data Collection

Data collection at the sites will be performed via paper format or electronic remote data capture by the Investigator or designee at the clinical sites using CRFs. Electronic data capture will be performed on validated systems only.

The database has limited access only to authorized individuals and provides an audit trail for the generation, modification and/or deletion of records.

The database is validated according to the FDA Guidance 'General Principles of Software Validation' and compliant with 21 CFR Part 11.

13.0 Financing and Insurance

The study will be sponsored by Epigenomics and Epigenomics will contract appropriate insurance to safeguard subjects participating in the study.

14.0 Publication Plan

The involved institution, Principal Investigators and Epigenomics have the right to make publicly available the results of their research and are encouraged to do so. Epigenomics will support publication of the study results, in cooperation with collaborating Institutions and Principal Investigators. Epigenomics will own all right, title and interest in and to any data, information and results generated solely by Epigenomics or its collaboration partners a) prior to the effective date of contractual agreement or b) independently of the samples provided by Institution (“Epigenomics Data”). Institution and Principal Investigator will not publish Epigenomics data, in whole or in part, without the written consent of Epigenomics.

Details of the publication(s) will be agreed upon in a steering committee comprised of members representing collaborating investigators, and Epigenomics. The steering committee will select collaborating investigators for authorship of the publication(s), and the order of authors of the publication(s) based on respective contribution to the Study. Following the joint publications referred to above, results generated during the study substantially based on the samples provided by Institution may also be jointly published.

15.0 Supplements

NA

16.0 References

1) Model F, Osborn N, Ahlquist D, Gruetzmann R, Molnar B, Sipos F, Galamb O, Pilarsky C, Saeger H-D, Tulassay Z, Hale K, Mooney S, Lograsso J, Adorjan P, Lesche R, Dessauer A, Kleiber J, Porstmann B, Sledziewski A, Lofton-Day C. Identification and validation of colorectal neoplasia-specific methylation markers for accurate classification of disease. Mol Cancer Res. 2007. 5(2):153-63.

2) Lofton-Day C, Model F, Devos T, Tetzner R, Distler J, Schuster M, Song X, Lesche R, Liebenberg V, Ebert M, Molnar B, Grützmann R, Pilarsky C, Sledziewski A. DNA methylation biomarkers for blood-based colorectal cancer screening. Clin Chem. 2008;54:414-23

3) Grützmann R, Molnar B, Pilarsky C, Habermann JK, Schlag PM, Saeger HD, Miehlke S, Stolz T, Model F, Roblick UJ, Bruch H-P, Koch R, Liebenber V, deVos T, Song X, Day RH, Sledziewski AZ, Lofton-Day C. Sensitive detection of colorectal cancer in peripheral blood by Septin 9 DNA methylation assay. PLoS ONE. 2008. 3(11): 1-8.

4) deVos T, Tetzner R, Model F, Weiss G, Schuster M, Distler J, Steiger KV, Grützmann R, Pilarsky C, Habermann JK, Fleshner PR, Oubre BM, Day R, Sledziewski AZ, Lofton-Day C. Circulating methylated SEPT9 DNA in plasma is a biomarker for colorectal cancer. 2009. Clin Chem. 55(7): 1337-46.

5) Rösch T. and Weiss G. Potential of a new blood test for colorectal cancer screening – The Septin 9 gene biomarker. European Oncol, 2010. May: 1-4.

6) Draft Guidance for Industry, Clinical Investigators, and Food and Drug Administration Staff ‘Design Considerations for Pivotal Clinical Investigations for Medical Devices’ (August 15, 2011), U.S. Department of Health and Human Services
